# Intact microstructure of the right corticostriatal pathway predicts creative ability in healthy adults

**DOI:** 10.1002/brb3.1895

**Published:** 2020-10-15

**Authors:** Farzaneh Rahmani, Hossein Sanjari Moghaddam, Mohammad Hadi Aarabi

**Affiliations:** ^1^ Department of Radiology Washington University in St. Louis St. Louis MO USA; ^2^ NeuroImaging Network (NIN) Universal Scientific Education and Research Network (USERN) Tehran Iran; ^3^ Faculty of Medicine Tehran University of Medical Sciences Tehran Iran

**Keywords:** corticostriatal pathway, creativity, diffusion MRI, diffusion‐weighted imaging

## Abstract

**Introduction:**

Creativity is one of the most complex functions of the human brain. The corticostriatal pathways have been implicated in creative thinking, yet few studies have addressed the microstructural underpinnings of creative ability, especially those related to the corticostriatal dopaminergic circuitry. We hypothesized that performance in creativity tests can be predicted based on diffusion metrics of the corticostriatal pathways and basal ganglia.

**Methods:**

A total of 37 healthy adults were included. Neuropsychological tests of creativity, including the alternative uses task (AUT), test of creative imagery abilities (TCIA), remote associates test (RAT), and creative achievement questionnaire (CAQ), as well as diffusion MRI data were acquired for each participant.

**Results:**

We demonstrated an independent effect of TCIA originality and TCIA transformativeness subscores, and RAT score in predicting the mean diffusivity (MD), mean axial diffusivity (AD), mean fractional anisotropy (FA), and mean generalized FA of the right corticostriatal pathway. We also observed independent effects of AUT elaboration subscore in predicting the AD of the right substantia nigra, and radial diffusivity (RD) of the right globus pallidus.

**Conclusion:**

Our results put a further spin on the “creative right brain” notion and question the presence of high‐creative and low‐creative networks in the brain.

## Graphical abstract

In this original paper, we hypothesized that performance in creativity tests can be predicted based on microstructural integrity of the corticostriatal pathways and basal ganglia based on diffusion MRI.

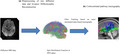

## 1 INTRODUCTION

Creativity has been defined both as a trait and state. Creativity as a trait is also referred to as creative potential, which can potentially predict creativity as a state or creative achievement (Jauk et al., 2014; Runco & Jaeger, 2012). Neuropsychologic batteries developed to assess trait creativity often evaluate the ability for disengagement and divergent thinking, as in the test of creative imagery abilities (TCIA), finding alternative uses for objects, as in the alternative uses task (AUT), or less commonly the ability for associative and convergent thinking, as required in the remote associates test (RAT; Guildford et al., 1978; Jankowska & Karwowski, 2015; Lee et al., 2014). State creativity is often quantified based on real‐life creative achievements using the qualitative creative achievement questionnaire (CAQ; Carson et al., 2005). Famously, creative achievement has been correlated with creative trait and openness to experiences, but not with academic achievement or intelligence (Jauk et al., 2013; Kéri, 2011).

Divergent and convergent thinking are considered central in definition of creative ability and are often used interchangeably and as a result of metacognitive *flexibility* and *persistence* (Hommel & Colzato, 2017; Zhang et al., 2020). According to Boot et al., creative cognition is a trade‐off and a direct result of cognitive flexibility, which is the ability to consider different approaches and divergent thinking, and cognitive persistence, which is the ability to sustain attention on cognitive problems in order to find solutions or in other words engaging in convergent thinking (Boot et al., 2017). It is also safe to assume that these two processes rely on different neural underpinnings as explained by a theory that defines distinct roles of mesocortical and mesolimbic dopaminergic pathways and frontostriatal dopaminergic modulations in creative flexibility and creative persistence. Where higher dopamine content in the mesocortical dopaminergic pathway, which connects the ventral tegmental area mainly to the prefrontal cortex, is in favor of cognitive persistence and convergence, higher dopamine levels in the nigrostriatal pathway benefits creative flexibility and divergence (Boot et al., 2017). Others have reported that divergent thinking and creative fluency are associated with increased activity in the lateral prefrontal cortex activity (Kleibeuker et al., 2017) and higher grey matter volume of the prefrontal cortex and striatum, (Jauk et al., 2015; Takeuchi et al., 2010a, 2010b). Moreover, higher activity of the frontostriatal circuitry is shown to be correlated with better convergent thinking ability (Abraham et al., 2018), implying that the creative performance owes to a balanced and correlated activation in both pathways. The role of frontostriatal circuitry in cognitive ability is further exemplified by the fact that the striatal network is involved in adaptive flexibility required during complex learning tasks (Kehagia et al., 2010), while the prefrontal cortical network alone is engaged in problem‐solving which requires persistence and thoroughness in creative thinking (Boot et al., 2017). Recently it was shown that diffusion metrics and connectivity of basal ganglia can predict creative ability in terms of divergent thinking, using the Abbreviated Torrance Test for Adults (Sunavsky & Poppenk, 2020). White matter integrity of left basal ganglia was a more strong predictor of generalized creative ability (Sunavsky & Poppenk, 2020). To date, no study has investigated the association of diffusion metrics and connectivity of basal ganglia subregions with creativity metrics.

Herein we investigated the association between conventional diffusion metrics (Ghazi Sherbaf et al., 2018; Mayeli et al., 2018; Mohajer et al., 2019) as well as diffusion MRI connectometry derived quantitative anisotropy (QA) of the corticostriatal pathway, the striatum and basal ganglia with creative ability and creative achievements in a group of young adults from the “Leipzig Study for Mind‐Body‐Emotion Interactions” (LEMON) cohort (Babayan et al., 2019; Mendes et al., 2019). We hypothesized that individual's creative ability and creative achievements is independently predicted by diffusion metrics of the corticostriatal pathway, the striatum and basal ganglia.

## 2 METHODS

### 2.1 Study design and participants’ characteristics

Data used in this study, including physiological, psychological, and neuroimaging records, were obtained from the LEMON database (Babayan et al., 2019) (http://doi.org/10.15387/fcp_indi.mpi_lemon). LEMON is a dataset of 3 Telsa MRI, EEG, cognitive tests, physiological parameters, and psychiatric symptoms gathered from 227 healthy adults in Leipzig, Germany, between 2013 and 2015. Potential subjects were all evaluated on Day 0 at the Day Clinic for Cognitive Neurology of the University Clinic Leipzig and the Max Planck Institute for Human and Cognitive and Brain Sciences (MPI‐CBS) in Leipzig, Germany where those with any history of neuropsychiatric disorders, hypertension, cardiovascular disorders, certain medications, illegal drugs and MRI contradictions, were excluded in the prescreening step (see Table 1 in Babayan et al., 2019). The study was carried out in agreement with the Declaration of Helsinki and the study protocol was approved by the ethics committee at the medical faculty of the University of Leipzig (reference number 154/13‐ff). Informed written consent was obtained from each participant.

**TABLE 1:** Demographic features of the study population

Age	26.17 ± 4.2^a^	BDI	4.8 ± 5.16	
Education	30 Gymnasium, 4 Realschule and 2 Hauptschule	CAQ	1.94 ± 0.92	
Sex	23 male, 13 female	AUT fluency	4.69 ± 1.52	
TCIA vividness	1.12 ± 0.32	AUT creative quality	2.8 ± 0.6	
TCIA originality	0.55 ± 0.30	AUT elaboration	0.88 ± 0.52	
TICA transformation	0.87 ± 0.29	AUT originality	0.036 ± 0.016	
		RAT	6.09 ± 2.17	

	MD	AD	FA	RD
Red nucleus, left	0.000666 ± 0.00002	0.00098477 ± 0.00004	0.41473 ± 0.035	0.000506273 ± 0.00003
Red nucleus, right	0.000646 ± 0.00002	0.00093947 ± 0.00003	0.40513 ± 0.036	0.000491703 ± 0.00003
Substantia nigra, left	0.000736 ± 0.00006	0.00109427 ± 0.00011	0.41863 ± 0.044	0.000561777 ± 0.00006
Substantia nigra, right	0.000706 ± 0.00003	0.00110757 ± 0.00010	0.46423 ± 0.065	0.00050272 ± 0.00004
Subthalamic nucleus, left	0.000626 ± 0.00004	0.00086917 ± 0.00005	0.40493 ± 0.040	0.000505133 ± 0.00004
Subthalamic nucleus, right	0.000636 ± 0.00003	0.00090287 ± 0.00006	0.41853 ± 0.050	0.000499235 ± 0.00004
Striatum, left	0.000866 ± 0.00007	0.00104227 ± 0.00007	0.20583 ± 0.010	0.000773784 ± 0.00007
Striatum, right	0.000786 ± 0.00007	0.00095107 ± 0.00007	0.20723 ± 0.010	0.000698237 ± 0.00006
Globus pallidus externa, left	0.000666 ± 0.00002	0.00095817 ± 0.00005	0.38463 ± 0.028	0.000516625 ± 0.00002
Globus pallidus externa, right	0.000696 ± 0.00003	0.00093257 ± 0.00005	0.31753 ± 0.024	0.000573539 ± 0.00002
Globus pallidus interna, left	0.000626 ± 0.00003	0.00104317 ± 0.00007	0.52513 ± 0.054	0.000422346 ± 0.00003
Globus pallidus interna, right	0.000646 ± 0.00003	0.00099347 ± 0.00007	0.44813 ± 0.052	0.000471353 ± 0.00003

Abbreviations: AD, axial diffusivity; AUT elaboration, amount of detail given to each answer; AUT fluency, the total number of given answers per subject; AUT originality, statistical rareness of the answers; AUT, alternative uses task; BDI, Beck Depression Inventory; CAQ, creative achievement questionnaire; Education, highest grade finished; FA, fractional anisotropy; MD, mean diffusivity; RAT, remote associates test (mean correct answers); RD, radial diffusivity; TCIA originality, the creative quality in terms of novel and surprising drawings; TCIA transformativeness, the level of modification and improvement of the initially generated idea; TCIA vividness, the level of detail and abstraction of the drawing; TCIA, test of creative imagery abilities.

^a^ All data are expressed as mean ± standard deviation.

Following prescreening, subjects were invited to Max Planck Institute for Human Cognitive and Brain Sciences (MPI‐CBS) to acquire structural MRI and diffusion‐weighted imaging (DTI) on a 3 Tesla scanner (MAGNETOM Verio, Siemens Healthcare GmbH; Appointment 1). Also, on their first appointment demographic information including age, sex, and education status were extracted, and all subjects answered to the Beck Depression Inventar‐II (BDI) via a home‐based online tool. On fifth appointment subjects responded to a set of neuropsychiatric questionnaires, including creativity tests that were administered using pen‐and‐paper. Importantly, although correlation of academic achievement with creativity has shown inconsistent results (Kéri, 2011), we used subjects education status as a potential covariate in partial correlation analyses and regression models (Gymnasium, Realschule, and Hauptschule). Also, as studies have pointed to an inverse association between severity of depressive and creativity (Miller et al., 2019; Taylor, 2017) we included participant depression scores as covariates of partial correlation analyses and regression models. Subjects enrolled in the current study consisted of 37 healthy native German young adults (23 males, mean age: 26.17 ± 4.2 years) for whom both the diffusion MRI (DMRI) and neuropsychiatric tests of interest were available.

### 2.2 Neuropsychological assessment

Neuropsychological assessments pertaining to creative ability including those assessing divergent thinking; TCIA and AUT, those assessing convergent thinking; RAT and creative achievements; CAQ, as well as the Beck Depression Inventar‐II were performed on the second visit of all participant. In tests were the final score was calculated a mean of scores from separate judges –TCIA and AUT‐ judges were all psychologists or psychiatry students specialized or trained in tests of creativity.

#### 2.2.1 Test of creative imagery abilities

The TCIA is a measure of creative imagery abilities using a drawing task (Jankowska & Karwowski, 2015). Seven ambiguous figures are presented and participants are requested to complete them in a creative way. The drawings are rated by five trained judges in three different categories: (a) vividness, which describes the level of detail, (b) originality, which refers to degree of novelty, and (c) transformativeness, which refers to the degree improvement of the initial idea of the drawings. The interrater reliability between the judges using intra‐class correlation was 0.73–0.76.

#### 2.2.2 Alternative uses task

Alternative uses task is a measure of divergent thinking ability (Guildford et al., 1978). Participants are requested to write down novel and creative uses for three items. Two minutes are given to create and write down the ideas and two top answers for each item are marked by the participant. Created items were assessed by three trained judges who rated the answers regarding (a) creative quality, (b) elaboration of details in each item. Subjects will also receive a fluency score based on the total number of answers given, as well as an originality score based on statistical rareness of their responses. Statistical rareness calculated based on an inverse correlation with the frequency of each answer. Semantically similar ideas were counted as one. Interrater reliability using intra‐class correlation was 0.74–0.82.

#### 2.2.3 Remote associates test

The German version of RAT consisting of 20‐word puzzles was used to measure creativity in terms of convergent thinking and problem‐solving (Lee et al., 2014). Each word puzzle in RAT contains three seemingly unrelated stimulus words and participants are requested to find out a unifying fourth word related to each of the three words. A total of 40 s is given for each puzzle (30 s thinking time and ten seconds answering time).

#### 2.2.4 Creative achievement questionnaire

Participants answered to the German version of CAQ which evaluates the level of creative achievements (i.e., creative state) in ten different domains including visual arts, dance, music, drama, culinary arts, architecture, creative writing, humor, science, and invention (Carson et al., 2005). Each domain consists of eight ranked questions qualitatively rating the magnitude of the creative achievements.

#### 2.2.5 Beck Depression Inventar‐II

The BDI‐II (Beck et al., 1961) is a subjective assessment of depressive symptoms in adolescents and adults. All subjects answered to the German version of BDI based on their feelings and symptoms in the two weeks prior to the assessment (Hautzinger et al., 1995). The inventar consists of 21 multiple‐choice items describing statements about depressive states in a four‐point Likert scale.

### 2.3 Image acquisition

Diffusion tensor imaging (DTI) data were acquired on a 3 Tesla scanner (MAGNETOM Verio, Siemens Healthcare GmbH) equipped with a 32‐channel head coil. The diffusion MRI data were collected using a multi‐band accelerated sequence and an in‐plane GRAPPA. Seven b0 image and 60 diffusion‐weighted images were acquired. The imaging parameters were as follows: *b*‐value = 1,000 s/mm^2^, TR = 7,000 ms, TE = 80 ms, GRAPPA acceleration factor = 2, bandwidth = 1,502 Hz/Px, field of view = 220 × 220 mm^2^, imaging matrix = 128 × 128 and voxel size = 1.7 × 1.7 × 1.7 mm^3^.

### 2.4 DTI ROI analysis

Diffusion‐weighted imaging images were corrected for head motion and eddy current artifacts using ExploreDTI toolbox. Recently it has been shown that diffusion metrics of basal ganglia is associated with overall creative ability in healthy adults (Sunavsky & Poppenk, 2020), although no study has evaluated the association of these metrics with different creativity traits at a subregional level. ExploreDTI tool for correction of echo planar imaging (EPI) distortions is integrated with the subject motion and eddy current distortions correction. In this way, all corrections are performed in one interpolation step to minimize blurring effects. The undistorted modality will be used to unwarp the deformations in the diffusion data which would then be resampled to the space of the undistorted modality. We used a nonrigid registration to map the diffusion‐weighted data to ATAG MNI Basal Ganglia map and for all corrections (subject motion, eddy currents and EPI). To define regions of interest (ROI), we performed automated ROI analysis with the Atlasing of the basal ganglia (ATAG) tool (Keuken et al., 2014). The ATAG uses ultra‐high resolution 7T MRI to provide unprecedented levels of detail on structures of the basal ganglia in‐vivo and including probability maps of the striatum, globus pallidus externa, globus pallidus interna, red nucleus, substantia nigra, red nuclei and subthalamic nuclei. After these preprocessing steps, the generalized fractional anisotropy (GFA), fractional anisotropy (FA), mean diffusivity (MD), axial diffusivity (AD), and radial diffusivity (RD) values were calculated in the 12 brain regions mapped through the ATAG atlas. We also analyzed the volume of the left and right cerebrum and calculated the asymmetry index of cerebrum for each individual. The average and standard deviation of the cerebrum asymmetry indices were all under 1% (mean ± *SD* = 0.83 ± 0.83) indicating no significant asymmetries between left and right hemispheres. All procedures performed using the ExploreDTI toolbox.

### 2.5 Corticostriatal pathway tractography

The DMRI data were corrected for subject motion, eddy current distortions, and susceptibility artifacts due to the magnetic field inhomogeneity using ExploreDTI toolbox (Leemans et al., 2009). DMRI tractography analyses were performed using the software DSI Studio (http://dsi-studio.labsolver.org), which is publicly available. The preprocessed diffusion‐weighted data were reconstructed in the MNI space using q‐space diffeomorphic reconstruction (QSDR) to obtain the spin distribution function (SDF; Yeh et al., 2011). A diffusion sampling length ratio of 1.25 was used. The SDF is an estimate of the peak density of diffusion spins for any given voxel orientation (Yeh & Tseng, 2011). Quantitative anisotropy (QA) is then estimated based on SDF and used to build the connectivity matrix within the tract of interest. QSDR is a model free algorithm that builds a matrix of orientation function at different diffusing spins to quantify the density of water diffusion, in contrast to conventional diffusivity metrics, at different orientations of any given voxel.

**FIGURE 1:**
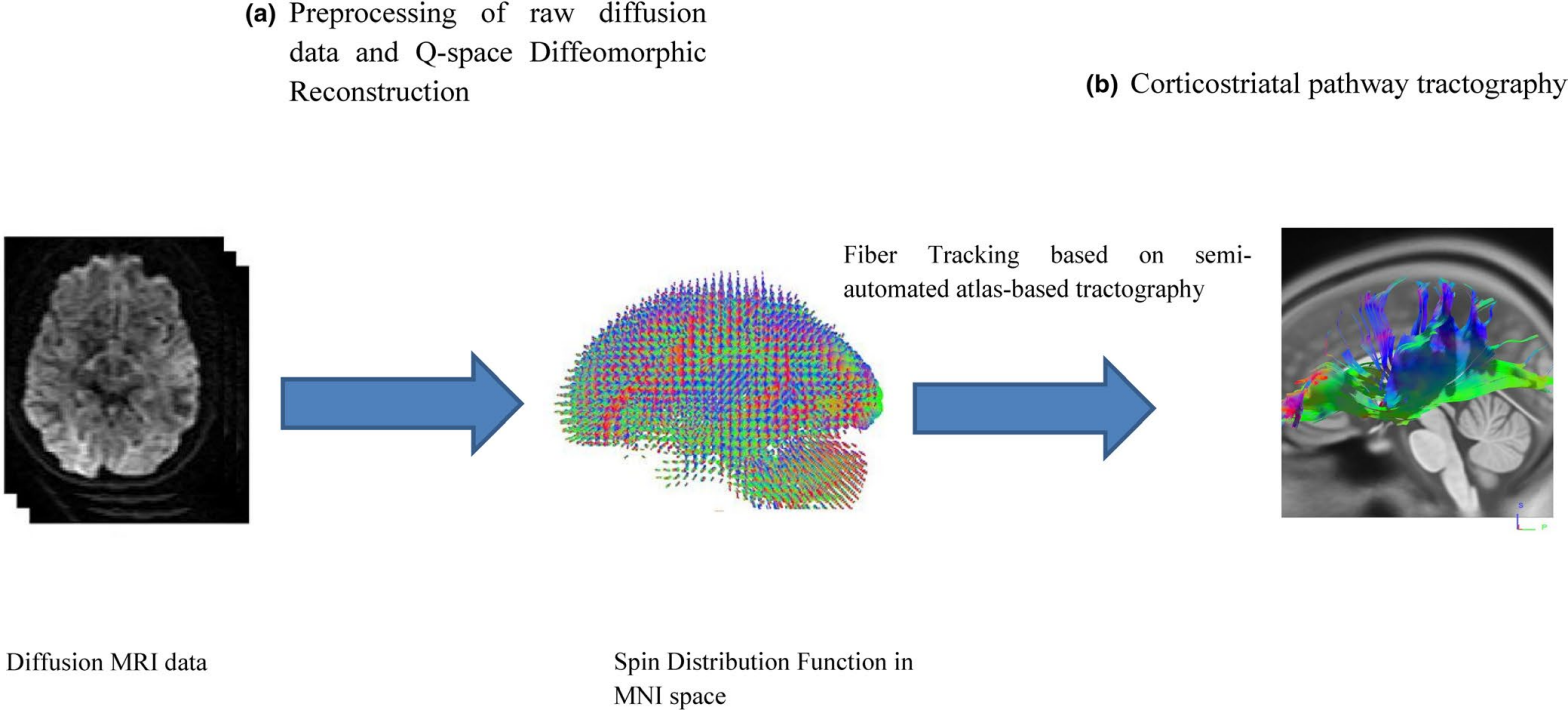
Overview of corticostriatal pathway tractography

We used the semi‐automated atlas‐based tractography based on the HCP842 tractography atlas (Yeh et al., 2018) and used the DSI Studio to calculate the Hausdorff distance between each track and compare it with those in the HCP842 tractography atlas (Yeh et al., 2018; Figure 1). The distance metric was defined based on the atlas proposed in Yeh et al. (2018) study. Based on the distance value, each track was then labeled by their closest track in the atlas and the tracks labeled by “corticostriatal pathway” were selected. The above‐mentioned process is provided in the current version of DSI Studio. The QA threshold was set at 0.2 and the angular threshold was 30°. Also, the step size was 0.5 mm, and tracks with a length shorter than 30 or longer than 300 mm were discarded. A total of 5,000,000 seeds were placed. The QA, FA, MD, AD and RD of bilateral corticospinal tracts were calculated using built‐in functions of the DSI studio.

### 2.6 Statistical analyses

Statistical analyses were performed using the SPSS20 software available under the shared license of the Tehran University of Medical Sciences. The correlation between diffusivity variables of 12 ROI and bilateral corticostriatal tracts, creativity scores, and age, were investigated using simple Pearson's correlation. Mann–Whitney *U* test was used to compare diffusometric variables between sex groups, and Kruskal–Wallis statistics was implemented to identify between group differences in study variables according to education status (Gymnasium, Realschule, and Hauptschule). Significant variables were then entered into a multiple regression model. We controlled the inflation in type‐I error due to multiple comparisons using the parametric bootstrap built‐in function of SPSS20 and as explained before (Westfall, 2011). The type‐I error rate was set at 5% for significant results.

We devised a multiple regression model to identify the independent association of diffusivity variables and significant creativity score, age, or sex, as identified through correlation analyses. First, the Durbin‐Watson statistic was used to check for independence of observations between regression models. To check for the presence of multicollinearity, variance inflation factor (VIF) was calculated which was lower than 10 for all independent variables indicating an absence of multicollinearity. To check for normality of residuals, the normality Q‐Q plots were drawn and visually inspected. Finally, the normality plot of residuals against predicted values in each model were visually inspected to check for homoscedasticity in variances in each model. The Stepwise or Enter methods were used interchangeably to identify the mode of best fit for each set of dependent and independent variables.

### 2.7 Ethical approval

All procedures performed here including human participants were in accordance with the ethical standards of the institutional research committee and with the 1964 Helsinki declaration and its later amendments or comparable ethical standards.

## 3 RESULTS

### 3.1 Association between demographic features, creativity, and diffusometric parameters

Demographic features and mean values of diffusometric variables are shown in Table 1. All but one subject were right‐handed. We performed simple Pearson's correlation test between diffusometric variables of 12 basal ganglia ROI and bilateral corticostriatal pathways, and creativity tests, including the TCIA, AUT, RAT, and CAQ total and subscores, BDI score, as well as age. An inverse correlation was found between participant's age and AUT fluency subscore (Pearson's *R* value = −.421, *p*‐value = .011). Higher BDI score (depressed mood), directly correlated with TCIA originality and AUT fluency subscores (Pearson's *R* value = .352, *p*‐value = .035; and Pearson's *R* value = .344, *p*‐value = .04; respectively). Higher BDI score was also associated with lower AD and GFA in the right corticostriatal pathway (Pearson's *R* value = −0.477, *p*‐value = .003; and Pearson's *R* value = .395, *p*‐value = .017; respectively). No difference was found in any of the neuropsychiatric test scores between males and females and among education subgroups (Gymnasium, Realschule, and Hauptschule).

### 3.2 Creative ability is independently associated with right dominant alterations in diffusion metrics of the corticostriatal pathway

We investigated the association between diffusometric variables of bilateral corticostriatal pathways and creativity scores, using a simple Pearson's correlation test. The results were controlled for participants BDI score and age according to the findings from simple correlation analyses described in Section 3.1. We identified significant correlations between diffusometric variables only in the right corticostriatal pathway and with the TCIA, AUT, and RAT scores (Table 2).

**TABLE 2:** Association of neuropsychological tests of creativity with diffusometric variables of corticostriatal pathway

Feature	TCIA originality	TCIA transformativeness	AUT fluency	RAT
Right mean diffusivity				
*R*	−.363	—	−.337^b^	—
*p*‐value	.03	—	.045	—
Right axial diffusivity				
*R*	−.330^a^	−.338^a^	—	—
*p*‐value	.049	.044	—	—
Right fractional anisotropy				
*R*	—	—	—	.372
*p*‐value		—	—	.026
Right generalized fractional anisotropy				
*R*	−.331^a^	−.364^a^	—	.406
*p*‐value	.049	.029	—	.014

Abbreviations: AUT fluency, the total number of given answers per subject; AUT, alternative uses task; *R*, Pearson’s correlation coefficient or standardized *β* value; RAT, remote associates test (mean correct answers); TCIA originality, the creative quality in terms of novel and surprising drawings; TCIA transformativeness, the level of modification and improvement of the initially generated idea; TCIA, test of creative imagery abilities.

^a^ The correlation between AD and GFA of the right corticostriatal tract and TCIA originality and TCIA transformativenss did not withstand controlling for BDI score.

^b^ The correlation between AD and GFA of the right corticostriatal tract and AUT fluency did not withstand controlling for participants age.

In the next step, we devised a multiple regression model to investigate the independent correlation of diffusivity measures of the right corticostriatal pathway with creativity scores. We chose pairs with significant correlation and tested the presumptions of the multiple regression model for each pair of dependent variable and factor (here diffusivity metrics in the right corticostriatal pathways), as explained in the methods section. We adopted the Enter and Stepwise methods alternatively to enter the factors in each regression model in order to investigate their independent association with creativity scores including TCIA originality, TCIA transformativeness, AUT fluency, and RAT. Age and BDI score were added to the regression model as factors where applicable, provided that the presumption of noncollinearity was fulfilled.

The multiple regression method showed a significant model that negatively predicted TCIA originality subscore by the independent effect of mean MD of the right corticostriatal pathway (*p*‐value: .03, *F*(1,34), *β*: −.363). In another model, TCIA transformativeness subscore was negatively predicted by mean AD (*p*‐value: .044, *F*(1,34), *β*: −0.338), and positively predicted by mean GFA (*p‐*value: .029, *F*(1,34), *β*: 0.364) of the right corticospinal pathway. Finally, higher RAT score was independently associated with higher mean FA and GFA of the right corticostriatal pathway (*p*‐value: .026, *F*(1,34), *β*: 0.372), and *p*‐value: .014, *F*(1,34), *β*: 0.406, respectively). All statistical analyses including simple and partial Pearson's correlation and multiple logistic regression had a type‐II error rate of <0.2.

### 3.3 Diffusivity in globus pallidus and substantia nigra independently predicts creative ability

We investigated the correlation between diffusometric variables of the bilateral striatum, globus pallidus externa, globus pallidus interna, subthalamic and red nuclei with creativity scores. Demographic features were entered in the model as cofactors where applicable. Presumptions of the multiple regression model were tested for each pair of dependent variable and factor, as explained in the methods section.

We identified a significant correlation between FA in the right red nucleus and AUT creative quality subscore (Pearson's *R* value = .451, *p*‐value < .01), and between FA of the right red nucleus with mean QA of bilateral corticostriatal pathways (Pearson's *R* value = −.400, *p*‐value = .016 for right, and Pearson's *R* value = −.370, *p*‐value = .027 for left). Entering these variables into a multiple regression model, neither AUT creative quality subscore nor QA of the corticostriatal pathways could independently predict FA of the right red nucleus, potentially due to significant multicollinearity between diffusometric variables (i.e., QA of the corticospinal pathway and FA of red nucleus).

Our results also indicated an indirect association between AD and MD of the right substantia nigra and AUT elaboration subscore (Pearson's *R* value = −.501, *p*‐value < .01, and Pearson's *R* value = −.455, *p*‐value < .01). There was also a significant difference in AD of the right substantia nigra between male and female participants. Entering these variables into a multiple regression model, significant and independent effects of AUT elaboration subscore (*p*‐value: .004, *F*(2,33), *β*: −.430) and sex (*p*‐value < .001, *F*(2,33), *β*: 0.537) were identified in predicting the AD of the right substantia nigra.

In a third model, RD of the right globus pallidus externa was significantly associated with AUT elaboration subscore (Pearson's *R* value = −.503, *p*‐value < .01), and with participant's age (Pearson's *R* value = −0.442, *p*‐value = .007). In a multiple regression model, both AUT elaboration subscore and age could independently predict the RD of the right globus pallidus externa (*p*‐value: .034, *F*(2,33), *β*: −0.322, and *p*‐value = .006, *F*(2,33), *β*: −0.426; respectively).

## 4 DISCUSSION

We examined the independent association between diffusivity measures of bilateral corticostriatal pathways, the striatum, globus pallidus externa, globus pallidus interna, subthalamic and red nuclei, with creative ability in a group of healthy young German adults. We identified: (a) an independent effect of TCIA originality subscore, TCIA transformativeness subscore, and RAT score in predicting the mean MD, mean AD, mean GFA, and mean FA of the right corticostriatal pathway, (b) independent effects of AUT elaboration subscore and sex in predicting the AD of the right substantia nigra, and (c) independent effects of AUT elaboration subscore and age in predicting the RD of the right globus pallidus. Together, our results suggest that creative ability is independently associated with alterations in diffusion metrics of the right corticostriatal pathway and basal ganglia in healthy young adults.

Our results indicated an independent negative association between mean MD and AD of the right corticostriatal pathways and the originality (novelty of ideas), and transformativeness (how well the subject has modified and improved the initial idea) in the TCIA test. As both MD and AD of the corticospinal pathways are shown to increase with age (Bennett et al., 2010; Gillespie et al., 2017), we added the subject's age as a covariate to the model. Given the fact that increase in MD and AD of white matter fibers indicates disruption of the microstructural organization and axonal myelin loss (Winklewski et al., 2018), our results suggest that creative ability in terms of divergent thinking is independently predicted by reduced AD and GFA of the right corticostriatal pathway in healthy adults. Moreover, we found an independent correlation between individual's ability in convergent thinking identified through RAT score, and higher FA and GFA of the right corticostriatal pathway. This was consistent with results of a previous report on the direct association between frontal cortical white matter integrity and higher creative ability (Takeuchi et al., 2010b). Importantly, the dominance of the right cerebral hemisphere in creative processing and associative thinking corroborates our results demonstrating a right dominant association between corticostriatal diffusivity and creativity scores in our study population (Aberg et al., 2017).

As the next step, we aimed to identify basal ganglial regions where diffusivity correlated with the individual's creativity scores. We identified a significant negative correlation between QA of the bilateral corticostriatal pathways and FA of the right red nucleus. Moreover, subject's ability to elaborate the details of the proposal object in the AUT task (AUT elaboration subscore), was correlated with the FA of the right red nucleus, yet the association did not survive the multiple regression model. It is important to address the inverse direction of correlation between bilateral corticostriatal QA and FA of the right red nucleus, as both variables (i.e., QA and FA) have been shown to be associated with myelin fiber integrity in the respective region or tract (Deleo et al., 2018; Yeh et al., 2016; Zhang et al., 2015). Indeed, connectivity measured by QA in diffusion MRI connectometry is shown to be analogous and highly correlated with FA measured in conventional DTI (Yeh et al., 2016). Red nucleus is primarily known for its role in cerebro‐cerebellar motor coordination (Belkhiria et al., 2017), receiving reciprocal projections to and from the ipsilateral motor and premotor cortices and superior frontal gyrus, as well as deep cerebellar nuclei in the dominant hemisphere (Cacciola et al., 2019; Habas & Cabanis, 2006; Lemon, 2016; Milardi et al., 2016). Increase in the QA of the red nucleus in the nondominant hemisphere is associated with a faster recovery of motor abilities following ischemic insult to the dominant corticospinal tract (Jang & Kwon, 2015; Kim et al., 2018). Moreover, an increase in the connectivity of the corticostriatal pathway is directly associated with goal‐directed behavior and development of value‐contingent performance during adolescent years (Insel et al., 2017). We, therefore, hypothesize that the inverse correlation between the FA of the right red nucleus and the QA of the corticostriatal pathway is a result of developmental emergence of goal‐directed movement patterns and maturation of the dominant corticostriatal pathway, along with simultaneous suppression of the nondominant motor axis (Cheng et al., 2017). In line with these explanations, another possibility would be the presence of multicollinearity between these two parameters and the AUT elaboration subscore underlies the correlation between the AUT elaboration subscore and the FA of the right red nucleus.

In another significant regression model, we identified a significant inverse correlation between AD of the right substantia nigra and AUT elaboration subscore independent of the subject's age and sex. AUT elaboration subscore could also predict the RD of the right globus pallidus independent of the effect of the subject's age. Lower AD and RD might indicate an association between increased myelination of the globus pallidus in association with creative ability. These results are also in line with the inverse correlation between mean diffusivity of bilateral substantia nigra and globus pallidus with creativity measured through divergent thinking (Takeuchi et al., 2017).

Previous studies have hypothesized a modulating role for the dopaminergic frontostriatal circuitry in facilitating creative cognition (Boot et al., 2017). Recently, however, it has been suggested that a highly connected network consisting of regions from three large‐scale brain networks, including the default mode, executive and salient networks, is chiefly responsible for the divergent thinking and creativity (Beaty et al., 2018). The mentioned study suggests that subcortical regions are engaged in a *low‐creative network* that facilitates habitual process and generation of ideas that are less likely to be regulated by input from default mode, executive, or salient networks (Beaty et al., 2018), networks that been attributed to divergent thinking and creativity (Shi et al., 2018; Takeuchi et al., 2011). In the context of the above findings, our results build upon the former hypothesis that creativity, as measured by divergent thinking, is associated with structural adaptations in the corticostriatal pathway and the basal ganglia, in particular, the substantia nigra and the globus pallidus.

## 5 CONCLUSION

Our results provide unprecedented evidence on the independent association between creative ability in terms of both divergent and convergent thinking with structural adaptations in the basal ganglia, in particular substantia nigra and globus pallidus, and corticostriatal pathways. Our evidence put a further spin on the “creative right brain” notion, as significant results were exclusively identified in right‐sided fibers/regions. The moderate intensity of the associations (beta value adopting values between 0.2 and 0.6) necessitates the use of a larger study population in future studies.

## CONFLICT OF INTEREST

The authors declare that they have no conflict of interest.

## AUTHOR CONTRIBUTIONS

F.R and M.H.A formulated, conceived, and designed the research. F.R and M.H.A preprocessed and analyzed the data. F.R and H.S.M and M.H.A wrote the manuscript.

### Peer Review

The peer review history for this article is available at https://publons.com/publon/10.1002/brb3.1895.

## Data Availability

Data used in this study including physiological, psychological, and neuroimaging records were obtained from the LEMON database (Babayan et al., 2019; Mendes et al., 2019) (https://openneuro.org/datasets/ds000221/versions/00002).
